# Supplementary material for: Remote inspection of adversary-controlled environments
Source: Nat Commun. 2023 Oct 17;14:6566. doi: 10.1038/s41467-023-42314-2 (PMC10582185; doi:10.1038/s41467-023-42314-2)
Supplement: Supplementary file 1 — Supplementary Information [file 41467_2023_42314_MOESM1_ESM.pdf]

Supplementary Information

Remote Inspection of Adversary-Controlled Environments

Johannes Tobisch, Sébastien Philippe, Boaz Barak, Gal Kaplun,
Christian Zenger, Alexander Glaser, Christof Paar, Ulrich Rührmair

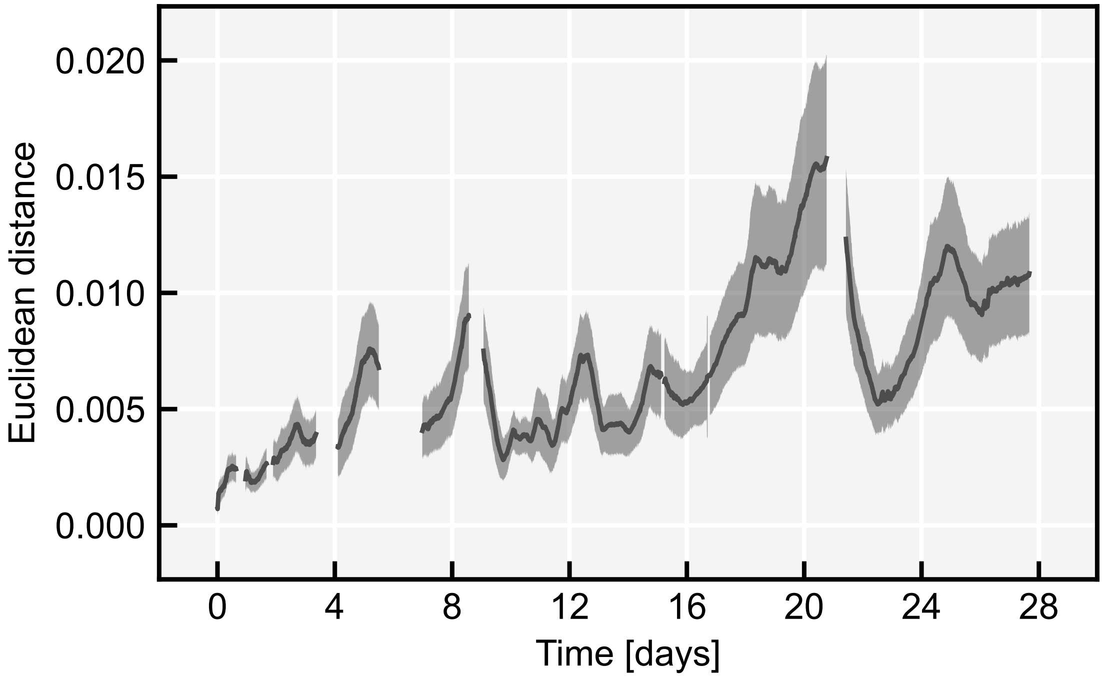

Supplementary Figure 1: Variance over time of challenge response pairs. Experimental data shows the time variance of challenge response pairs that occurred during the consecutive acquisition of all training sets. Gaps indicate periods during which no experimental data was collected (due to sanity checking and software updates). Time variation of the intra distance is estimated on the basis of 25 challenges that were measured in parallel with the training data. Shaded area indicates three times the standard deviation.

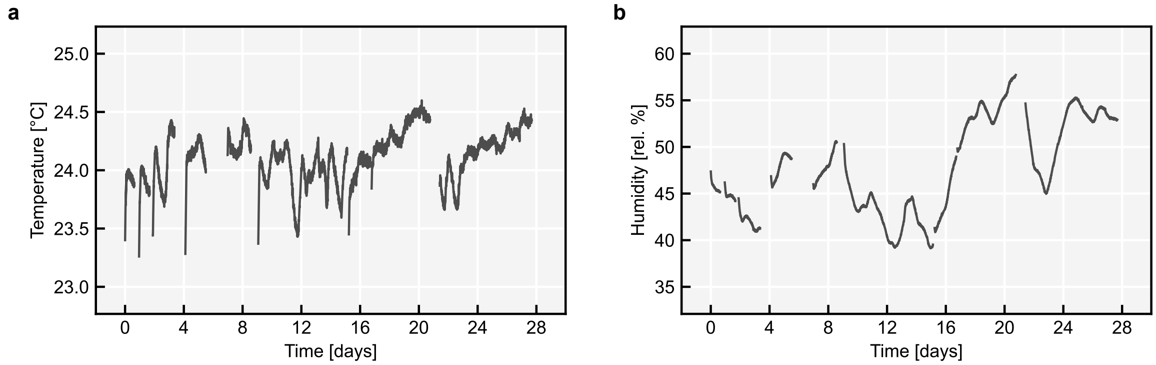

Supplementary Figure 2: Temperature and humidity drift. The range of temperature (**a**) and humidity (**b**) that was observed while capturing all training data sets. The temperature deviation is rather small (23.3°C – 24.6°C) while the humidity varied in a comparatively wider range (39.1 rel.% – 56.7 rel.%).

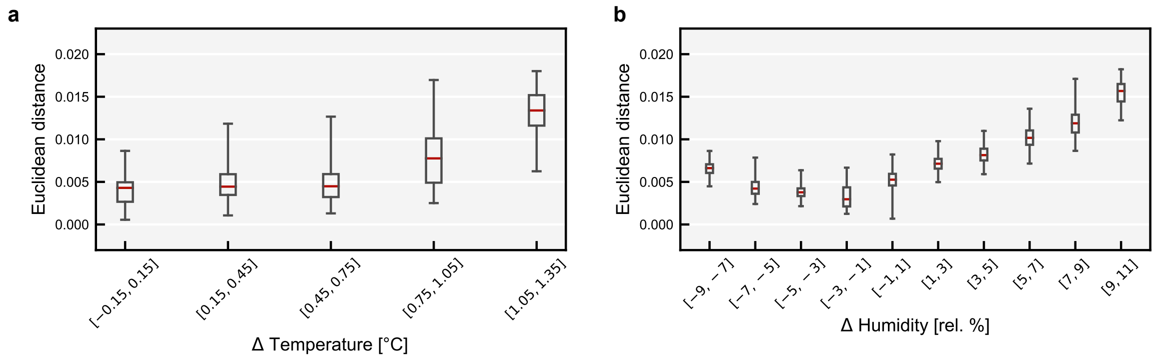

Supplementary Figure 3: Dependency between response variance and environmental drift. Intra distances are binned according to the temperature (**a**) and humidity (**b**) deviation from the reference measurement. The median is given in red, the whiskers show the extent of 99.73% of the data.

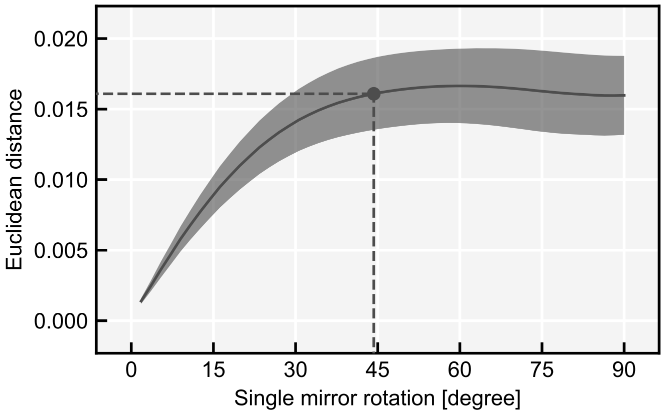

Supplementary Figure 4: Detecting minute physical changes in the room. Effect of rotating a single mirror from a reference position. At a rotation of 44° the response is significantly decorrelated. A detection threshold (3σ from the intra distance mean) is shown as a dashed horizontal line. Mean and standard deviation (shaded areas) were obtained from measurements over 100 challenges.

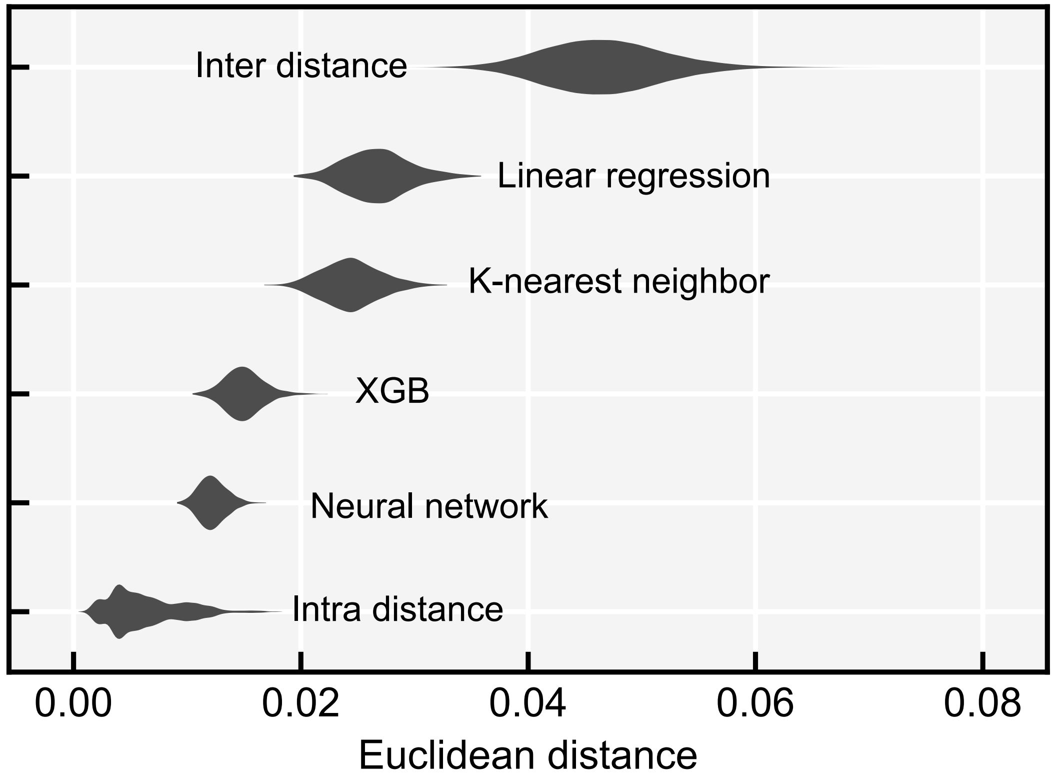

Supplementary Figure 5: Machine learning algorithm comparison. Comparison of the prediction error of different algorithms for 12 active mirrors. The models were trained using 1,279,000 challenge-response pairs and evaluated on a separate test set of 1,000 examples.

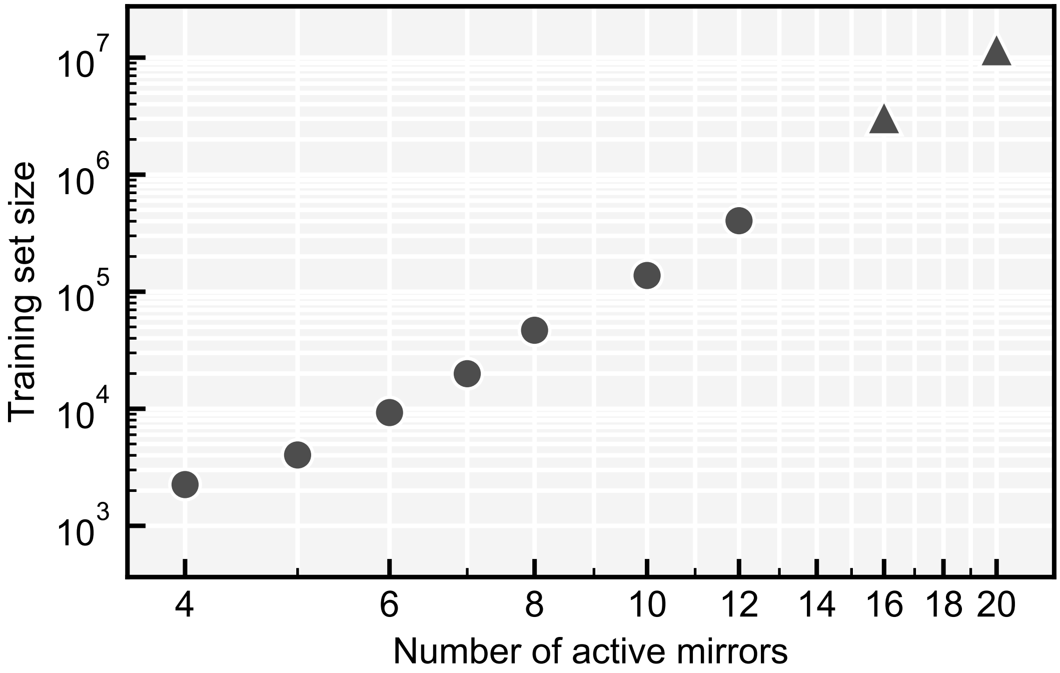

Supplementary Figure 6: Machine learning complexity scaling. The training set size at which the mean test error falls below the intra distance mean plus 3σ per number of active mirrors. Both axes are log-scaled.

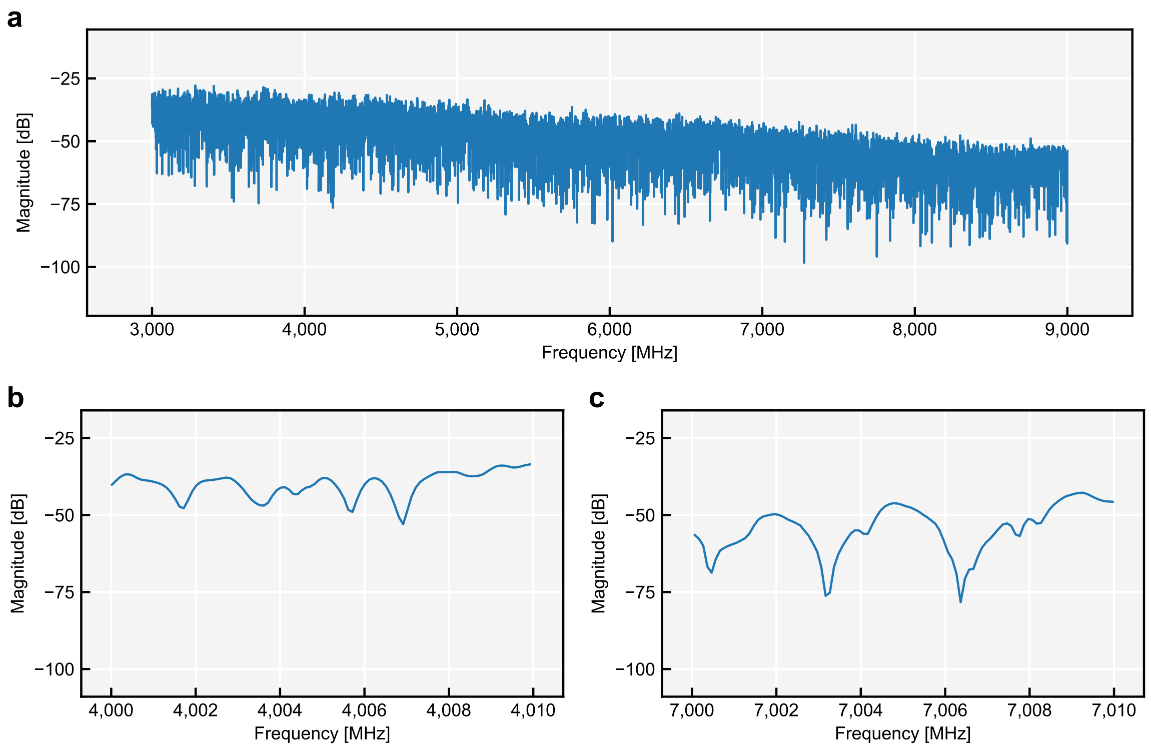

Supplementary Figure 7: Fine-grained response spectrum. Response spectrum for a single challenge (total spectrum in **a**, excerpts in **b** and **c**). Spectrum was sampled at 60,000 points between 3 GHz and 9 GHz. It is clearly visible that small changes in the frequency in the range of ~ 1 MHz lead to significant changes in the magnitude of the transmission coefficient (cf. Supplementary Fig. 8). These small-scale fading effects are a sign of a highly-reflective environment that provides a rich multipath environment.

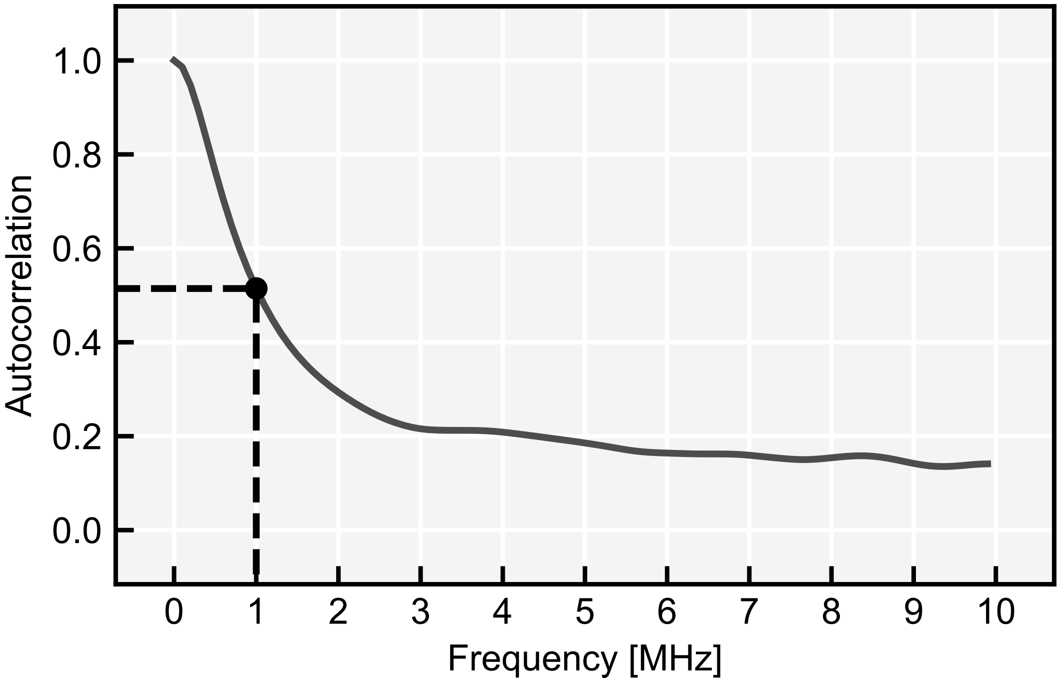

Supplementary Figure 8: Coherence bandwidth determined by autocorrelation. The mean of the autocorrelation of the response spectrum over 100 random challenges. Spectrum was sampled at 60,000 points between 3 GHz and 9 GHz. A shift of the response by ~ 1 MHz results in a fall of the correlation below 0.5.
